# Molecular detection of Torque teno virus in different breeds of swine

**DOI:** 10.1186/1743-422X-8-503

**Published:** 2011-11-03

**Authors:** Zhiwei Wu, Hongning Wang, Xin Yang, Zhongbing Guan, Yingshun Zhou

**Affiliations:** 1 School of Life science, Sichuan University, Animal Disease Prevention and Food Safety Key Laboratory of Sichuan Province,"985"Project Science Innovative Platform for Resource and environment Protection of Southwestern, Key Laboratory of Bio-resources and Eco-environment of Ministry of Education, Chengdu, Sichuan, 610065, P.R. China

**Keywords:** nested polymerase chain reaction (nested PCR), serums, swine breeds, Torque teno virus (TTV)

## Abstract

**Background:**

Torque teno virus (TTV), of the *Anelloviridae *family, *Iotatorquevirus *genus, is a non-enveloped, single-stranded, and negative sense DNA (ssDNA) virus infecting human and many domestic animals including swines. Very little information is known about the investigations of TTV prevalence in different swine breeds so far.

**Methods:**

In this study, 208 serum samples collected from seven swine breeds (*Rongchang pig*, *Chenghua pig*, *Zibet pig*, *Wild boar*, *Duroc*, *Landrace*, *Large Yorkshire*) from two independent farms were detected to determine the prevalence of two swine TTV genogroups, TTV1 and TTV 2, by nested polymerase chain reaction methods, and to analyse prevalence difference among these breeds.

**Results:**

The results showed that the prevalence of TTV in the seven breeds was 92%-100%. No significant difference (p > 0.05) in TTV infection was observed between different breeds. Interestingly, significantly higher prevalence for TTV1 in *Rongchang *boars (90%) and for TTV2 in *Rongchang *sows (95%) were detected, while co-infection rate (43.8%) was lower than other breeds. Sequence analysis showed that the homology of TTV1 and TTV2 were over 90.9% and 86.4% in these breeds, respectively.

**Conclusions:**

The results indicated that TTV was widely distributed in the seven swine breeds. The prevalence of both TTV genogroups associated with swine breeds and genders. This study also respented the first description of swine TTV prevalence in different swine breeds. It was vitally necessary to further study swine TTV pathogenicity.

## Background

Torque teno virus (TTV) was first found from a Japanese patient with post-transfusional hepatitis of unknown etiology (non-A-G) in 1997 [1]. TTV is a small, non-enveloped, single-stranded, negative sense, and circular DNA virus, belonging to the *Anelloviridae *family, *Iotatorquevirus *genus [2,3]. TTV is frequently detected in humans and swines, but its pathogenicity/virulence and its ability to induce specific diseases are currently unknown [4,5]. However, TTV co-infection with other virus species could be related with such a disease but to date no definitive correlation has been found with any disease syndrome [6-10]. The swine TTV genogroup 2 has been found to be more common in pig suffering from postweaning multisystemic wasting syndrome (PMWS), a disease caused by porcine circovirus type 2 (PCV2), than in non-infected pigs. Recent reports showed that porcine TTV partially contributed to inducing PMWS, porcine reproductive and respiratory syndrome (PRRS), porcine dermatits and nephriopathy syndrome and hepatitis of pigs infection [5,11-15]. Furthermore, the same family virus, Torque teno sus viruses (TTSuV), infection has been confirmed with cases of PCV2 infection, PRRSV infection but no research has been proved a contribution of the TTSuV in such conditions [11-13].

Torque teno virus (TTV) is able to infect several vertebrate species, including human, swine, chicken, sheep, cattle, dogs, and cats [16,17]. Analysis of genomic DNA reveals a well-conserved genomic organization among the various TTV species [3,18]. However, the TTVs that infect different vertebrate species show different genome lengths and a great variability in the sequences. The genome ranges from 2.1 kb to 3.8 kb in size. The genomes of TTVs infecting humans and chimpanzees range from 3.7 to 3.9 kb in size, those infecting pigs and dogs are 2.8 to 2.9 kb in length, respectively, and the TTV with the smallest genome identified to date, 2.1 kb, has been detected in the cat [3]. TTV contains 3 or 4 partially overlapping open reading frames (ORF1, ORF2, ORF3 and/or ORF4) translated from negative ssDNA as well as a short stretch of untranslated region (UTR) with high GC content [17]. Nested PCR amplifications of the conserved regions in the UTR of TTV genogroup 1 and TTV genogroup 2, respectively, have been widely used [19].

The widespread prevalence of TTV in swine has been found in many countries, including USA, Canada, China, Thailand, Korea, Italy, Franc and Spain, varying between 24% and 100% by the nested PCR methods [20-23]. Detection of the European wild boars (sus scorfa) indicates a high prevalence of TTV [24]. In addition, TTV have been described in a large number of species [17,25], and multiple infections of porcine TTV with distinct genotypes or subtypes was found in the same pig [4,26].

The goal of this study was to detect, using species-specifc UTR nested PCR methods, the presence of TTV genogroup 1 and 2 in serum samples of swines of seven breeds from different farms, genders and age groups, and to further insight on its epidemiological characteristics.

## Results

In this study, the results from TTV nested PCR detection in serum samples from seven breeds of pig were summarized in Table 1. In all detected serum samples, TTV1 or TTV2, TTV1, TTV2, and co-infected with both TTV genogroups were for 96.2% (200/208), 77.9% (162/208), 82.2% (171/208), and 65.4% (136/208) for nested PCR positive, respectively. No significant difference on the prevalence between TTV1 and TTV2 was observed in all serum samples tested (*p *> 0.05). Prevalence difference between both TTV genogroups was mainly caused by the factors of swine breeds, and seondly for genders, age groups, and farms.

**Table 1:** Prevalence of TTV genogroups in different breeds of pig

Farm	Breed	Prevalence of swine TTV genogroup							
		
		TTV1 or TTV2		TTV1		TTV2		TTV1 and TTV2	
		
		Positive	Prevalence	Positive	Prevalence	Positive	Prevalence	Positive	Prevalence
A	Chenghua pig(n = 25)	23	92.0%	20	80.0%	19	76.0%	18	72.0% a, b
	Rongchang pig(n = 38)	37	97.4%	26	68.4%	25	65.8%	16	42.1% a,
	Wild boar(n = 20)	19	95.0%	15	75.0%	16	80.0%	12	60.0% a, b
	Zibet pig(n = 10)	10	100%	9	90.0%	10	100%	9	90.0% b

Total	n = 93	89	95.7%	70	75.3%	70	75.3%	55	59.1%

B	Landrace (n = 25)	24	96.0%	21	84.0%	23	92.0%	20	80.0% a, b
	Large Yorkshire(n = 16)	16	100%	13	81.5%	15	93.4%	13	81.5% a, b
	Rongchang pig(n = 42)	39	92.9%	29	69.0%	31	73.8%	19	45.2% a
	Duroc (n = 32)	32	100%	29	90.6%	32	100%	29	90.6% b

Total	n = 115	111	96.5%	92	80.0%	101	87.8%	81	70.4%

Number of analyzed serum samples from different swine breeds in each farms (n), total amount of positive animals, prevalence of in percentage, different farms (A, B), and different letters (a, b, c) within the same column mean statiscal significant differences in different breeds and sources TTV prevalence when comparing one breed/source to another.

In the seven breeds of pig, the prevalence of TTV1 or TTV2 ranged from 92% to 100%. The TTV1 and TTV2 co-infection was between 43.8% and 90.6%. For TTV1, infection rates ranged from 68.8% to 90.6%, and between 70% and 100% for TTV2 (Table 1). In the same breed, TTV2 prevalence was higher than TTV1 except *Chenghua *pig, and no significant difference on the TTV prevalence between these breeds was observed (ρ > 0.05).

**Figure 1:**
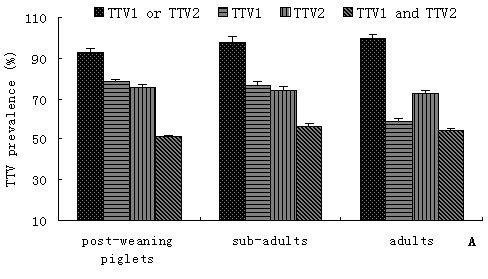
**Mean prevalence of TTV genogroup 1 or TTV genogroup 2, TTV genogroup 1, TTV genogroup 2, and co-infection of both TTV geongroups in swines from different age classes**.

**Figure 2:**
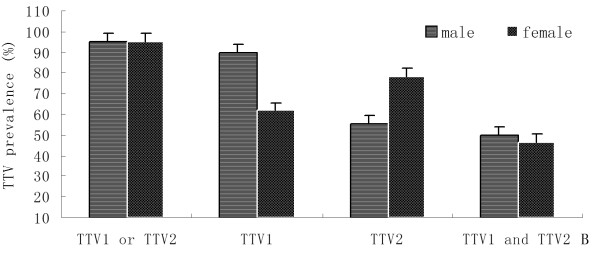
**Mean prevalence of TTV genogroup 1 or TTV genogroup 2, TTV genogroup 1, TTV genogroup 2, and co-infection of both TTV geongroups in *Rongchang *swines from different sexes**.

**Figure 3:**
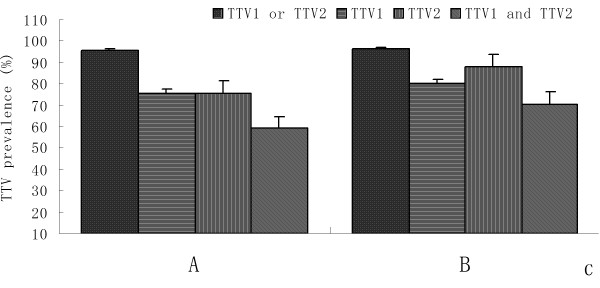
**Mean prevalence of TTV genogroup 1 or TTV genogroup 2, TTV genogroup 1, TTV genogroup 2, and co-infection of both TTV geongroups in the seven swine breeds from different farms**.

In addition, prevalence of both TTV genogroups were analyzed according to the age classes, genders and farms of residence of pigs. The swine breed was the main contributors to the variation in prevalence observed among different age groups, genders and farms. Infection rates of TTV1 or TTV2 in post-weaning piglets, sub-adults, and adults were 93.3% (98/105), 98.3% (58/59), and 100% (44/44), repectively. Results showed that significantly more post-weaning piglets tested were positive for TTV1, but infection rates of TTV2 was higher in the adults (Figure 1). In general, the difference in prevalence of both TTV genogroups was not significant between sexes in the seven breeds. For TTV1, prevalence in boar and sows populations ranged from 60% (3/5 in Yorkshire) to 100% (6/6 in Zibet pig), 61.7% (37/60 in Rongchang pig) to 100% (14/14 in Duroc), respectively. For TTV2, prevalence in boar and sows populations were between 45% (9/20 in Rongchang pig) and 100% (6/6 in Zibet pig, 18/18 in Duroc, 5/5 in Large Yorkshire), 78.6% (11/14 in Chenghua pig) and 100% (4/4 in Zibet pig, 14/14 in Duroc, 12/12 in Landrace, 11/11 in Large Yorkshire), respectively (Table 2). An exception was found in the Rongchang swine, which the infection rates were significantly higher for TTV1 in Rongchang male pigs (90%, 18/20) and for TTV2 in Rongchang female pigs (95%, 47/60), while co-infection rates (40% for male, 45% for female) was lower than the other breeds (Figure 2). In different farms, no significant difference of TTV infection was observed according to the Table 1. Positive rates of TTV, TTV1, TTV2, and co-infection of TTV1 and TTV2 in farm A were higher than in farm B (Figure 3).

**Table 2:** Prevalence of TTV genogroups in different sexes and breeds of swines

gender	Breed	Prevalence of swine TTV genogroup							
		
		TTV1 or TTV2		TTV1		TTV2		TTV1 and TTV2	
		
		Positive	Prevalence	Positive	Prevalence	Positive	Prevalence	Positive	Prevalence
Male	Rangchang pig(n = 20)	19	95.0%	18	90.0%	9	45.0%	8	40.0%
	Chenghua pig(n = 11)	11	100.0%	9	81.8%	8	72.7%	7	63.6%
	Zibet pig(n = 6)	6	100.0%	6	100.0%	6	100.0%	6	100.0%
	Wild boar(n = 8)	7	87.5%	6	75.0%	5	62.5%	4	50.0%
	Duroc pig(n = 18)	18	100.0%	15	83.3%	18	100.0%	15	83.3%
	Landrace pig (n = 13)	12	92.3%	12	92.3%	11	84.6%	11	84.6%
	Yorkshire pig (n = 5)	5	100.0%	3	60.0%	5	100.0%	3	60.0%

Female	Rangchang pig(n = 60)	57	95.0%	37	61.7%	47	95.0%	27	45.0%
	Chenghua pig(n = 14)	12	85.7%	11	78.6%	11	78.6%	10	71.4%
	Zibet pigB(n = 4)	4	100.0%	3	75.0%	4	100.0%	3	75.0%
	Wild boarC(n = 12)	12	100.0%	9	75.0%	11	91.7%	8	66.7%
	Duroc pig(n = 14)	14	100.0%	14	100.0%	14	100.0%	14	100.0%
	Landrace pig (n = 12)	12	100.0%	9	70.2%	12	100.0%	9	70.2%
	Yorkshire pig (n = 11)	11	100.0%	10	90.9%	11	100.0%	10	90.9%

Number of analyzed serum samples from different breeds and genders in different sources (n), total amount of positive animals (Positive) and prevalence of in percentage (Prevalence).

Analysis of the obtained sequences from the seven breeds of pig revealed a lower genetic diversity within genogroup variant, but a high genetic diversity between genogroups. The pair wise comparison of nucleotide sequences within a genogroup showed high percentage of homology (90.9%-99.2% among TTV1, 86.4%-100% among TTV2) (Table 3 and Table 4). The percentage of sequence identity among different variants was not obviously associated with swine breeds. Diversity between genogroup 1 and 2 sequences was high, showing overall sequences identities of only 42-56%. Comparison of TTV sequences from the breeds of swine with that of Genbank reported revealed high identities on average, over 91% and 94% for TTV1 and TTV2, respectively. Genetic distances were similar to the percentage identity value between the seven swine breeds.

**Table 3:** Homoloy analysis of TTV1 UTR sequence

list	Zibet pig	Chenghua pig	Duroc	Landrace	Large Yorkshire	Rongchang pig	Wild boar
Zibet pig	100	95.1	97.0	93.5	95.8	92.4	92.8
Chenghua pig	95.1	100	93.5	93.2	92.8	92.0	92.4
Duroc	97.0	93.5	100	92.0	94.3	91.3	91.3
Landrace	93.5	93.2	92.0	100	92.8	91.6	99.2
Large Yorkshire	95.8	92.8	94.3	92.8	100	95.8	92.0
Rongchang pig	92.4	92.0	91.3	91.6	95.8	100	90.9
Wild boar	92.8	92.4	91.3	99.2	92.0	90.9	100

**Table 4:** Homology Analysis of TTV2 UTR sequence

list	Zibet pig	Chenghua pig	Duroc	Landrace	Large Yorkshire	Rongchang pig	Wild boar
Zibet pig	100	87.4	88.3	86.4	88.3	88.3	89.3
Chenghua pig	87.4	100	96.3	93.0	97.6	96.3	94.1
Duroc	88.3	96.3	100	91.6	98.1	100	92.6
Landrace	86.4	93.0	91.6	100	91.2	91.6	92.6
Large Yorkshire	88.3	97.6	98.1	91.2	100	95.8	94.1
Rongchang pig	88.3	96.3	100	91.6	95.8	100	92.6
Wild boar	89.3	94.1	92.6	92.6	94.1	92.6	100

## Discussion

The presence of TTV is increasingly reported around the world, particularly in human and swine [12,27]. The present study represented the first description of TTV genogroups infection in different breeds of pig populations by serum samples detected using the nested PCR methods in China. Results indicated a high prevalence of TTV genogroup 1 (77.9%) and 2 (82.2%) in the seven breeds of pigs, similar to that of other regions swines [12]. No significant differences of TTV prevalence were observed in different breeds. However, Zhu et al [28] reported that the prevalence of TTV1 and TTV2 were at 8.57% and 6.03%, respectively, no pigs were found to be infected with both TTV1 and TTV2 in chinese swine herds by stools analysis. Moreover, in many countries TTV genogroup 2 has higher prevalence than genogroup 1[16], while in this study all infection rates of both TTV genogroups were very high, and no significant difference was observed between the TTV1 and TTV2. Thus, it may be inferred that, besides swine breeds, TTV prevalence could be caused by surroundings, conditions of farms, and variation of the "virues".

To date, two species-specific TTV genogroups, TTV1 and TTV2, has been described in swine [18]. The extent of nucleotide sequence variation between both TTV genogroups depended greatly on the region of the viral genome analysed [12,20]. In this study, the sequencing results showed that TTV nucleotide similarity in different porcine breeds were 92% to 96% and 91% to 100% comparing with porcine TTV sequences obtained in the Genbank for TTV genogroup 1 and 2, respectively. Therefore, in the seven swine breeds, it is not found new TTV genogroups or clusters.

Interestingly, many studies reported that TTV genogroup 2 prevalence was very high in female swines [4,12,20,23]. In this study, the result was not only further confirmed, but also we found high prevalence of both TTV genogroups existed in male swines. In both sexes, co-infections of both TTV genogroups were very higher in the other breeds except *Rongchang pig*. But one study found that co-infection of both TTV genogroups was not observed in swine stools detected using regular PCR methods [28]. By analyzing the phenomena, it might be caused by the factors, such as different detected samples and methods, physiological functions and micro-ecological conditions of the swine breeds. Some studies has reported that changes of gut microbiome may be associated with bowel diseases or obesity [29].

In humans, TTV has been detected in faeces, saliva, semen, sera, umbilical cord blood and aborted, and slaughterhouse collected foetuses, suggesting both horizontal and vertical transmission as routes of viral dissemination [17,20,30-32]. Also, swine TTV has been found in sera, plasma, faeces and veterinary vaccines, and the results indicated faecal-oral transmission and injection of vaccines as the most significant ways of transmission [33,34]. Recent studies reported that TTV was also distributed in swine tissues included brain, lung, mediastinal and mesenteric lymph nodes, heart, liver, spleen, kidney and bone marrow in different ages [35]. In this study, prevalence of TTV genogroups detected was higher in serum samples of the adults than in the young swines, indicating that vertical transmission by blood contributed to the spread of virus. This result further confirmed the fact that swine TTV can be transmitted vertically through sow-to-piglet [31].

According to the different farms, TTV prevalences of seven swine breeds were also analyzed. In the farm A and B, no significant differences were observed in all serum samples detected. However, if swine feeding and living conditons would effect on TTV prevalence, the same swine breed, *Rongchang pig*, which lived in the two independent farms, was detected. The results showed that the conditions were not influence on TTV prevalence in *Rongchang pig*. This indicated that difference of TTV infection was caused by swine breed. Furthermore, sequence analysis indicated that homology of TTV was very high (over 91%) in the seven swine breeds. Therefore, the theory of horizontal or vertical transmission of TTV was further supported by evidences obtained in the study.

## Conclusion

The results of the present study showed that TTV prevalence were ubiquitous distribution in different swine breeds, and the breeds were one of these factor caused TTV prevalence difference. While many studies found that PMWS and PRRSV infections in swine populations were associated with prevalence of TTV genogroup 2 and 1, respectively [12,15,36]. Therefore, further studies on the detailed pathogenicity, prevention, treatment and diagnosis of TTV in pig populations, including the relationship of the virus with PMWS, PRRS, and other swine diseases, are needed.

## Materials and methods

Serum samples were collected between April and December in 2009 at two independent farms, which are about 300 km apart and are carried out the nearly same management models and feeding program, and artificially insemination were taken in swine production system (table 1). A total of 208 serum samples of seven breeds of swine (*Rongchang pig*, *Zibet pig*, *Chenghua pig*, and *wild boar*; *Duroc*, *Landrace*, and *Large Yorkshire*), which were randomly collected from age groups (post-weaning piglets [1-2 month of age], n = 105; sub-adult pigs [3-6 months of age], n = 59; and adults [over 12 months of age], n = 44), and gender (female, n = 127; male, n = 81), were detected to obtain an overview of the presence of TTV using UTR nested PCR methods. About 10 to 42 serum samples were collected per studied breed. All of the animal experiments in the study were healthy and were carried out in accordance with the guidelines of Sichuan Province on the Review of Welfare and Ethics of Laboratory Animals, and under the protocol (SCU-AM-2010-01221) approved by the Animal Ethics Committee of Sichuan University.

Blood samples were centrifuged at 1500 × g for 10 min at 4°C, and obtained sera were stored at -80°C prior to usage. Viral DNA was extracted from 200 μl of the serum samples using the QIAamp DNA Blood Mini Kit (Qiagen, China). The entire extraction process were performed according to the manufacturer's instructions and the DNA was eluted in 50 μL elution buffer. Presence of TTV genogroup 1 or TTV genogroup 2 in serum samples was determined using previously described nested PCR methods [19], the reaction and amplification conditions were slightly modified from previously described systems. For TTV genogroup 1, first round 25 μL PCR reactions contained 5 μL of serum DNA, 20 pmol primer forward-1 (5'-CGG GTTCAGGAGGCTCAAT-3') and reverse-1 (5'-GCCATTCGGAACTGCACTTAC T-3'), 2.5 mM dNTP, 2 mM MgCl and 0.5 U Taq DNA polymerase (Promega, USA). The amplification was initiated by heating for 5 min at 94°C, followed by 35 cycles of 30 s at 94°C, 30 s at 54°C, 40 s at 72°C and a final extension for 5 min at 72°C. Then, 5 μL of the serum amplification product was used as the template for nested PCR using primers pair forward nested-1(5'-CTCGCTTCGCTCGCACCAC-3') and reverse nested- 1(5'-CAGTTTACTGGGA ACGCCCTAATTCT-3') 20 pmol each primer, 2.5 mM dNTP, 2 mM MgCl and 0.75 U Taq DNA polymerase initiated for 5 min at 94°C, followed by 35 cycles of 25 s at 94°C, 25 s at 56°C, 30 s at 72°C and a final extension for 5 min at 72°C. For TTV genogroup 2, the amplification step was carried out as described above using primers pairs forward-2 (5'-AGTTACACATAACCACCAAACC-3') and reverse-2(5'-ATTACCGCCTGCCCGATAGG C-3') for the first round and forward nested-2 (5'-CCAAACCACAGGAAACTGTGC-3') and reverse nested-2(5'-CTTGACTCCGCTCTCAGGAG-3') for the nested PCR. The first round amplification was initiated by heating for 5 min at 94°C, followed by 35 cycles of 25 s at 94°C, 25 s at 52°C, 30 s at 72°C and a final extension for 5 min at 72°C. The nested PCR amplification was performed as above except the annealing temperature was changed to 55°C. The second round amplification products of TTV genogroup 1 and TTV genogroup 2 were analyzed using electrophoresis on a 2% (w/v) agarose gel and were visualized with Sybrsafe (Invitrogen, Canada).

A total of 14 nested PCR positive amplicons (one TTV1 and one TTV2 were chosen per breed) were randomly selected and sequenced to validate the nested PCR amplification results and to analyze the similarities among TTV genogroups of different breeds. The 14 products amplified were excised from 1% agarose gel and purified using the QIAquick PCR Purification Kit (Qiagen) according to the manufacturer's instructions. Sequencing reaction were done using Big Dye Terminator v3.1 cycle sequencing kit (Biosystem) and ran with ABI Prism 3100 sequence analyzer (Perkin-Elmer). Nucleotide sequences were edited using VectorNTI and aligned with partial loaded sequence of the swine TTV UTR in Genbank nucleotide database using the WU_BLAST 2.0 programs.

Statistical analysis were carried out for detected amounts of TTV infection in seven breeds of pig among the different breeds, farms, age groups, and genders using SPSS software. Level of statistical significance was fixed at a P value < 0.05.

## Abbreviations

**TTV**: Torque teno virus; **TTV1**: Torque teno virus group 1; **TTV2**: Torque teno virus group 2; **ssDNA**: single-stranded, and negative sense DNA; **nested PCR**: nested polymerase chain reaction; **PCV2**: porcine circovirus type 2; **PMWS**: postweaning multisystemic wasting syndrome; **PRRS**: porcine reproductive and respiratory syndrome; **TTSuV**: Torque teno sus viruses; **ORF**: open reading frames; **UTR**: untranslated region.

## Competing interests

The authors declare that they have no competing interests.

## Authors' contributions

ZW carried out the design of the study, most of the experiments and wrote the manuscript. HW participated in revising the manuscript and the experimental design. XY, ZG, and YZ participated in acquisition, analysis and interpretation of data. All authors read and approved the final manuscript.
